# Neuroprotective Effect of IRL-1620, an Endothelin B Receptor Agonist, on a Pediatric Rat Model of Middle Cerebral Artery Occlusion

**DOI:** 10.3389/fped.2018.00310

**Published:** 2018-10-23

**Authors:** Enrique G. Cifuentes, Mary G. Hornick, Suresh Havalad, Ramona L. Donovan, Anil Gulati

**Affiliations:** ^1^Advocate Children's Hospital, Park Ridge, IL, United States; ^2^Department of Pharmaceutical Sciences, Chicago College of Pharmacy, Midwestern University, Downers Grove, IL, United States

**Keywords:** stroke, cerebral blood flow, endothelin B receptor agonists, ischemic penumbra, cerebral infarction, reperfusion

## Abstract

**Objective:** The purpose of this study was to determine the potential neuroprotective effect of endothelin B (ET_B_) receptor agonist IRL-1620 treatment in a pediatric model of ischemic stroke.

**Design:** A prospective, animal model study.

**Setting:** An experimental laboratory.

**Subjects:** Three-month-old male Wistar Han rats.

**Interventions:** The rats underwent permanent middle cerebral artery occlusion (MCAO). At 2, 4, and 6 h post MCAO, they were treated with saline, IRL-1620 (5 μg/kg, IV), and/or ET_B_ antagonist BQ788 (1 mg/kg, IV).

**Measurements and Main Results:** The rats were evaluated over the course of 7 days for neurological and motor deficit, cerebral blood flow (CBF), and infarct volume. Young rats treated with IRL-1620 following MCAO improved significantly in neurological and motor assessments as compared to the vehicle-treated group, as measured by neurological score (*P* = 0.00188), grip test (*P* < 0.0001), and foot-fault error (*P* = 0.0075). CBF in the infarcted hemisphere decreased by 45–50% in all groups immediately following MCAO. After 7 days, CBF in the infarcted hemisphere of the IRL-1620 group increased significantly (*P* = 0.0007) when compared to the vehicle-treated group (+2.3 ± 23.3 vs. −45.4 ± 10.2%). Additionally, infarct volume was significantly reduced in IRL-1620-treated rats as compared to vehicle-treated rats (*P* = 0.0035, 41.4 ± 35.4 vs. 115.4 ± 40.9 mm^3^). Treatment with BQ788 blocked the effects of IRL-1620.

**Conclusions:** IRL-1620 significantly reduced neurological and motor deficit as well as infarct volume while increasing CBF in a pediatric rat model of cerebral ischemia. These results indicate that selective ET_B_ receptor stimulation may provide a novel therapeutic strategy in the treatment of pediatric ischemic stroke as has been demonstrated in adult ischemic stroke.

## Introduction

Stroke is an increasingly recognized cause of morbidity and mortality in children, with an estimated annual incidence of 2.4 to 11 per 100,000 (1). Acute ischemic stroke accounts for 50% of strokes in children. Of those children that experience an acute ischemic stroke, 10–25% will die; 25% of surviving children will have a recurrence; and up to 66% will suffer neurological deficits, learning disabilities and developmental delays, and/or seizures. In the last decade, hospitalizations for acute ischemic stroke in children have risen partly as a result of increased risk factors, such as longer survival of children with cardiac anomalies, diabetes, hypertension, obesity, infection, trauma, sickle cell disease, cancer, hypercoagulable states, and metabolic disorders (2, 3). In children needing intensive care, the use of central venous catheters poses a significant risk for deep venous thrombosis; a patent foramen ovale in these patients creates the path and potential for thromboembolic stroke (4, 5).

Because of the complex pathology of ischemic stroke, treatment remains a challenge and currently includes only thrombolytic therapy. A safe and effective therapy that provides neuroprotection is urgently needed. Restoring cerebral blood flow (CBF) and saving dying neurons are goals for ischemic brain injury therapy. Since the discovery of increased endothelin (ET) concentration in both plasma and cerebrospinal fluid after cerebral ischemia, ET and its possible role in the pathophysiology of ischemic stroke has been the focus of much discussion. The biological action of ETs, a family of vasoactive peptides, is mediated by G-protein coupled receptors, ET_A_ and ET_B_. The human brain contains a high density of ET receptors, located throughout neurons, astrocytes, and cerebro-vasculature (6). These centrally located receptors regulate various functions, including CBF and cellular migration, proliferation, and apoptosis (7–9).

ET_B_ receptor subtype binding and functional studies have demonstrated glia mainly express ET_B_ receptors, whereas ET_A_ receptors are located mainly on neurons (10). ET_B_ receptors mediate relaxation of the small pial arteries and small arterioles via activation of endothelial nitric oxide synthase (11). The small pial arteries and arterioles penetrating the brain play a major role in the maintenance of CBF (auto-regulation).

IRL-1620 is a synthetic analog of endothelin-1 that activates ET_B_ receptors on vascular endothelium, resulting in the release of nitric oxide (NO) via nitric oxide synthase and causing vasodilatation, stimulation of CBF, enhancement of angiogenesis, and overall improving functionality after ischemic stroke (12–14). As a result of the promising preclinical data regarding the efficacy of IRL-1620 in treating ischemic stroke in the adult population, IRL-1620, also known as PMZ-1620, has recently been produced for human testing. A Phase I (CTRI/2016/11/007509) clinical trial determined that PMZ-1620 was well-tolerated and safe when administered, with a Minimum Intolerable Dose (MID) of 0.9 μg/kg and a Maximum Tolerated Dose (MTD) of 0.6 μg/kg (15). A Phase II (CTRI/2017/11/010654) clinical trial examining the effect of PMZ-1620 at a proposed therapeutic dose of 0.3 μg/kg for the treatment of ischemic stroke in adults is currently underway. Recent studies on the use of IRL-1620 to improve post-stroke function and limit the size of ischemic damage have reported promising results. Studies conducted in our lab have demonstrated that treatment with IRL-1620 in adult rats following mid-cerebral artery occlusion (MCAO) significantly reduces infarct volume by 70–80%, oxidative stress, and neurological deficit while increasing neurovascular remodeling (12–14). Recent investigations to determine the capacity of an injured brain to reorganize and restore itself following stroke have indicated that vascular and neuronal remodeling may be necessary components for functional recovery.

The purpose of this study was to determine whether IRL-1620 treatment would be neuroprotective in a pediatric model of ischemic stroke by specifically assessing whether IRL-1620 restores CBF and attenuates secondary damage in the penumbra.

## Materials and methods

### Subjects

Three-month-old male Wistar Han rats (corresponding to human age 8–16 years) weighing 250–300 g were allowed to acclimate to the testing environment for at least 4 days before use (16). The rats were housed in a room with controlled temperature (23 ± 1°C), humidity (50 ± 10%), and light (6:00 a.m. to 6:00 p.m.). Food and water were available continuously. Animal care and anesthetic and surgical procedures were approved by and conducted in compliance with the guidelines established by the Institutional Animal Care and Use Committee of Midwestern University (IACUC study approval MWU 2103). Animals were randomly divided into five groups with six rats per group. The groups received the following interventions.

Group I was the Sham group and received only vehicle (isotonic saline); rats in this group underwent a sham surgery wherein the right common and internal carotid arteries were isolated but no MCAO was performed. Group II was the MCAO + vehicle group; these rats underwent MCAO and received only vehicle. Group III was the MCAO + IRL-1620 group; rats in this group received ET_B_ agonist at 2, 4, and 6 h post MCAO. Group IV was the MCAO + BQ788 group; rats in this group underwent MCAO and received ET_B_ antagonist 15 min prior to vehicle administration. Finally, Group V was the MCAO + BQ788 + IRL-1620 group; rats in this group underwent MCAO and received the antagonist 15 min prior to the initial administration of the IRL-1620.

### Drugs

Anesthesia drugs: Ketamine (Henry Schein Animal Health, Dublin, OH, USA) at a dose of 100 mg/kg and xylazine (Lloyd Laboratories, Shenandoah, IA, USA) at a dose of 10 mg/kg were administered intraperitoneally (i.p.).

Study drugs: IRL-1620 (N-Succinyl-[Glu9, Ala 11,15] endothelin 1) (Bachem Americas, Inc., Torrance, CA, USA) was dissolved in isotonic saline and administered at a dose of 5 mcg/kg intravenously (i.v.) via tail vein at 2, 4, and 6 h post MCAO. BQ788 (American Peptide Co, Inc., Sunnyvale, CA, USA) was dissolved in isotonic saline and administered at a dose of 1 mg/kg i.v., 15 min prior to initial administration of either vehicle or IRL-1620 in Groups IV and V, respectively. The doses of both IRL-1620 and BQ788 were based on our preliminary studies (12, 13).

### Middle cerebral artery occlusion to induce focal cerebral ischemia

Induction of focal cerebral ischemia via MCAO was performed according to the method described by Koizumi et al. (17). Rats were anesthetized with ketamine (100 mg/kg, i.p.) and xylazine (10 mg/kg, i.p.). With the animal in a secured supine position, a midline incision was made and expanded to the right common carotid artery, exposing the internal and external carotid arteries. A 4.0 monofilament nylon thread (CP Medical, Portland, OR, USA) was used to occlude the middle cerebral artery. The nylon filament was advanced from the external carotid artery into the lumen of the internal carotid artery until a resistance was felt (~20 mm), indicating occlusion of the middle cerebral artery. The nylon filament was allowed to remain in place to create a permanent model of focal cerebral ischemia. In sham-operated animals, the common carotid artery and external carotid artery were exposed and the incision was sutured without touching the internal carotid artery.

### Neurological evaluation

Animals were subjected to a neurological evaluation prior to occlusion and at days 1, 4, and 7 post MCAO. The neurological evaluation was based on a 6-point scale, scored as follows: 0 = no deficits, 1 = failure to fully extend the left paw, 2 = circling to the left, 3 = paresis to the left side, 4 = no spontaneous walking, 5 = death (18). Animals were placed in an observation chamber for 15 min and scored according to performance.

### Motor performance tests

Four assessments were used to determine motor activity and coordination following MCAO. Animals were subject to blinded assessments prior to occlusion, and 1, 4, and 7 days post MCAO using a grip test, foot-fault test, rotarod, and spontaneous locomotor activity assessment.

#### Grip test

The device used for the grip test consisted of a string 50 cm in length pulled taut between two vertical supports and elevated 40 cm above a flat surface. The animal was placed on the string midway between the supports and evaluated according to a 6-point scale (19). The scoring was as follows: 0 = falls off, 1 = hangs on by two forepaws, 2 = hangs on by two forepaws and attempts to climb on, 3 = hangs on by 3+ paws, 4 = hangs on by all paws plus tail, and 5 = escapes.

#### Foot-fault error test

Animals were placed on an elevated grid floor with a mesh size of 30 mm for 1 min to acclimate. They were then observed for 1 min and evaluated for foot-fault errors (a misplaced limb falling through the grid) compared with paired steps as follows using the following equation (20):

% foot-fault error = (number of faults/number of paired steps) × 100.

#### Rotarod

Prior to MCAO, animals were acclimated to the rotating spindle of the rotarod apparatus (RotaRod 47700, Ugo Basile, Italy). They were placed on the rotating spindle, which was set to a constant 8 rotations per minute (RPM) until they demonstrated the ability to remain on the spindle for 60 s. Animals were then subject to a baseline acceleration trial (4-40 RPM) over 5 min. The acceleration trial was repeated at 1, 4, and 7 days post occlusion, and the time at which they fell off was recorded in seconds (21).

#### Spontaneous locomotor activity

Spontaneous locomotor activity was assessed using an animal activity meter (Opto-Varimex-4 Auto-Track System, Columbus Instruments, Columbus, OH, USA). Each animal was observed for a period of 10 min in a square enclosed area equipped with infrared photocells along the X, Y, and Z axes to quantitatively measure spontaneous horizontal and vertical motion.

### Cerebral blood flow

CBF in both cerebral hemispheres was monitored using Laser Speckle Contrast Analysis via the Pericam PSI High Resolution System (Perimed, Inc., Ardmore, PA, USA). The rats were anesthetized, and a paramedian incision was performed. The subcutaneous tissue and cranial fascia were dissected to reach the skull bone. The rats were then positioned precisely 10 cm below the laser with a 1.1 cm^2^ field centered over the midline. CBF was monitored at a frame rate of 25 images/s for 15 min before and at 1 h and 7 days post MCAO.

### Assessment of cerebral infarct volume

Following neurological and motor function testing on day 7 post MCAO, animals were euthanized by decapitation, and the brains were removed to determine infarct volume. The brains were quickly removed and chilled in saline at 4°C for 5 min. They were then cut into 2 mm thick coronal slices using a Brain Matrix (Harvard Apparatus, Holliston, MA, USA). Sections were incubated in 2% 2,3,5-triphenyltetrazolium chloride (TTC, Sigma, St Louis, MO, USA) dissolved in saline for 15 min at 37°C. The stained sections were then stored in 10% formalin and refrigerated at 4°C for further analysis. Infarct volumes were calculated by sampling each side of the coronal sections with a digital camera (Nikon, Melville, NY). The infarct area, outlined in white, was measured by image analysis software (Adobe Photoshop CS6, San Jose, CA, USA). The percent increase in size of the ipsilateral over the contralateral hemispheres was used to determine edematic swelling of the brain tissue (22). Infarct size is expressed as infarction volume in mm^3^, as the sum of infarct areas in each slice, corrected for edema.

### Statistical analysis

A power analysis was conducted using GraphPad Instat-3.1 (GraphPad Software, La Jolla, CA, USA). The power was set to 80% (beta = 0.8) and the level of significance (alpha) used was 0.05. The sample size in each group was 6 based upon expected change determined from results published in literature using similar procedures. Two-way analysis of variance followed by Tukey's *post-hoc* test was used to compare intergroup behavioral tests and CBF with considerations to time and treatment. One-way analysis of variance followed by Tukey's *post-hoc* test was used to compare infarct volume. A *p*-value of < 0.05 was considered to be significant. Analysis was completed using SPSS software (IBM, Armonk, NY, USA) and GraphPad Prism 7 (GraphPad Software, La Jolla, CA, USA). Data are presented as the mean ± standard deviation.

## Results

Results of all neurological, motor, and cerebral injury assessments for all time points and animal groups tested are presented in Table 1. Day 7 post MCAO reflected the maximum impact of IRL-1620 on recovery. Rats in Group III who received IRL-1620 had a significantly lower neurological deficit score (0.8 ± 0.4) in comparison to those in Group II or vehicle-treated rats (2.2 ± 0.7, *p* = 0.006). The IRL-1620-treated rats also had a significantly greater grip test scores (4.2 ± 0.7) when compared to vehicle- (1.5 ± 1.2, *p* = 0.004) and BQ877-treated animals (1.4 ± 1.4, *p* = 0.01). Foot fault error increased following MCAO in all groups as compared to Group I sham animals. BQ788 alone and in conjunction with IRL-1620 resulted in significantly greater error than the IRL-1620 group (Day7: 62.7 ± 39.0% and 65.1 ± 30.2% vs. 13.1 ± 12.4%, *p* < 0.0001 and *p* = 0.0014, respectively). Rotarod duration and distance traveled did not vary significantly between MCAO treatment groups.

**Table 1:** Results of all neurological, motor, and cerebral injury indices.

**Treatment groups**	**Time point (days)**	**Neurological deficit (6-point scale)**	**Grip test (6-point scale)**	**Foot-fault error (%)**	**RotaRod duration (seconds)**	**Distance traveled (cm)**	**Cerebral blood flow (% reduction)**	**Infarct volume (mm3)**
**I)** Sham Group	Baseline	0 ± 0	4.5 ± 0.8	9.0 ± 6.0	148.7 ± 37.7	3516.8 ± 550.2	0 ± 0	
	Day 1	0 ± 0	3.2 ± 0.8	5.1 ± 3.1	121.0 ± 32.1	2832.6 ± 562.8	−10.3 ± 22.3	
	Day 4	0 ± 0	3.2 ± 1.3	8.4 ± 5.9	131.6 ± 40.8	3532.6 ± 374.5		
	Day 7	0 ± 0	3.6 ± 0.5	5.5 ± 3.5	142.8 ± 41.9	4160.8 ± 1122.1	−7.7 ± 8.8	0 ± 0
**II)** MCAO + vehicle	Baseline	0 ± 0	4.4 ± 0.5	5.7 ± 5.3	125.9 ± 12.5	3768.4 ± 852.1		
	Day 1	2.0 ± 0.6*^*a*^*	2.4 ± 1.1	38.2 ± 17.0*^*a*^*	103.3 ± 37.9	1225.5 ± 664.1*^*a*^*	−49.6 ± 17.4*^*a*^*	
	Day 4	1.6 ± 0.5*^*a*^*	2.2 ± 1.1	31.2 ± 11.8	114.6 ± 50.4	2802.8 ± 613.3		
	Day 7	2.2 ± 0.7*^*a*^*	1.5 ± 1.2*^*a*^*	44.3 ± 25.7*^*a*^*	118.6 ± 55.4	2830.7 ± 660.5	−45.4 ± 10.2*^*a*^*	115.4 ± 40.9*^*a*^*
**III)** MCAO+ IRL-1620	Baseline	0 ± 0	4.6 ± 0.5	5.5 ± 6.7	112.6 ± 22.3	4231.8 ± 662.5		
	Day 1	1.7 ± 0.7	2.8 ± 0.7	28.7 ± 15.0	99.8 ± 103.4	904.8 ± 904.3*^*a*^*	−47.0 ± 18.8*^*a*^*	
	Day 4	1.2 ± 0.8	3.0 ± 1.4	25.9 ± 20.8	97.8 ± 45.0	1742.2 ± 1386.9*^*a*^*		
	Day 7	0.8 ± 0.4*^*c*^*	4.2 ± 0.7*^*c*^*	13.1 ± 12.4	144.2 ± 60.5	3361.0 ± 1563.6	2.3 ± 23.3*^*c*^*	41.4 ± 35.4*^*c*^*
**IV)** MCAO+ BQ788	Baseline	0 ± 0	3.8 ± 1.1	5.5 ± 4.1	97.2 ± 34.3	3948.8 ± 414.1		
	Day 1	3.0 ± 1.5*^*a*^^*b*^*	0.8 ± 1.1*^*a*^^*b*^*	73.2 ± 32.7*^*a*^^*b*^*	55.4 ± 79.8	957.4 ± 986.6*^*a*^*	−48.4 ± 15.3*^*a*^*	
	Day 4	2.8 ± 1.6*^*a*^^*b*^*	1.6 ± 1.6*^*b*^*	75.0 ± 22.5*^*a*^^*b*^*	37.4 ± 65.2	1551.6 ± 1587.3*^*a*^*		
	Day 7	2.8 ± 1.2*^*a*^^*b*^*	1.4 ± 1.4*^*a*^^*b*^*	62.7 ± 39.0*^*a*^^*b*^*	53.0 ± 70.3*^*b*^*	2366.8 ± 1346.6*^*a*^*	−31.7 ± 34.4*^*b*^*	93.9 ± 28.8*^*a*^^*b*^*
**V)** MCAO+IRL-1620+BQ788	Baseline	0 ± 0	4.7 ± 0.5	5.9 ± 5.0	146.0 ± 66.7	3668.3 ± 472.3		
	Day 1	3.0 ± 1.8*^*a*^^*b*^*	2.3 ± 1.9	49.4 ± 34.0	47.7 ± 38.6	382.3 ± 445.1*^*a*^*	−54.7 ± 22.5*^*a*^*	
	Day 4	2.3 ± 2.1*^*a*^*	1.7 ± 1.4*^*b*^*	50.0 ± 33.9	63.3 ± 92.0	1509.0 ± 1304.1		
	Day 7	2.3 ± 2.1*^*a*^^*b*^*	2.0 ± 1.5*^*b*^*	65.1 ± 30.2*^*a*^^*b*^*	83.0 ± 97.2	2099.3 ± 1879.9*^*a*^*	−53.1 ± 36.8*^*a*^^*b*^*	96.6 ± 10.1*^*a*^^*b*^*

a *p < 0.05 vs. Group I (Sham group)*,

b *p < 0.05 vs. Group III (IRL1620 group)*,

c *p < 0.05 vs. Group II (vehicle group)*.

**Figure 1:**
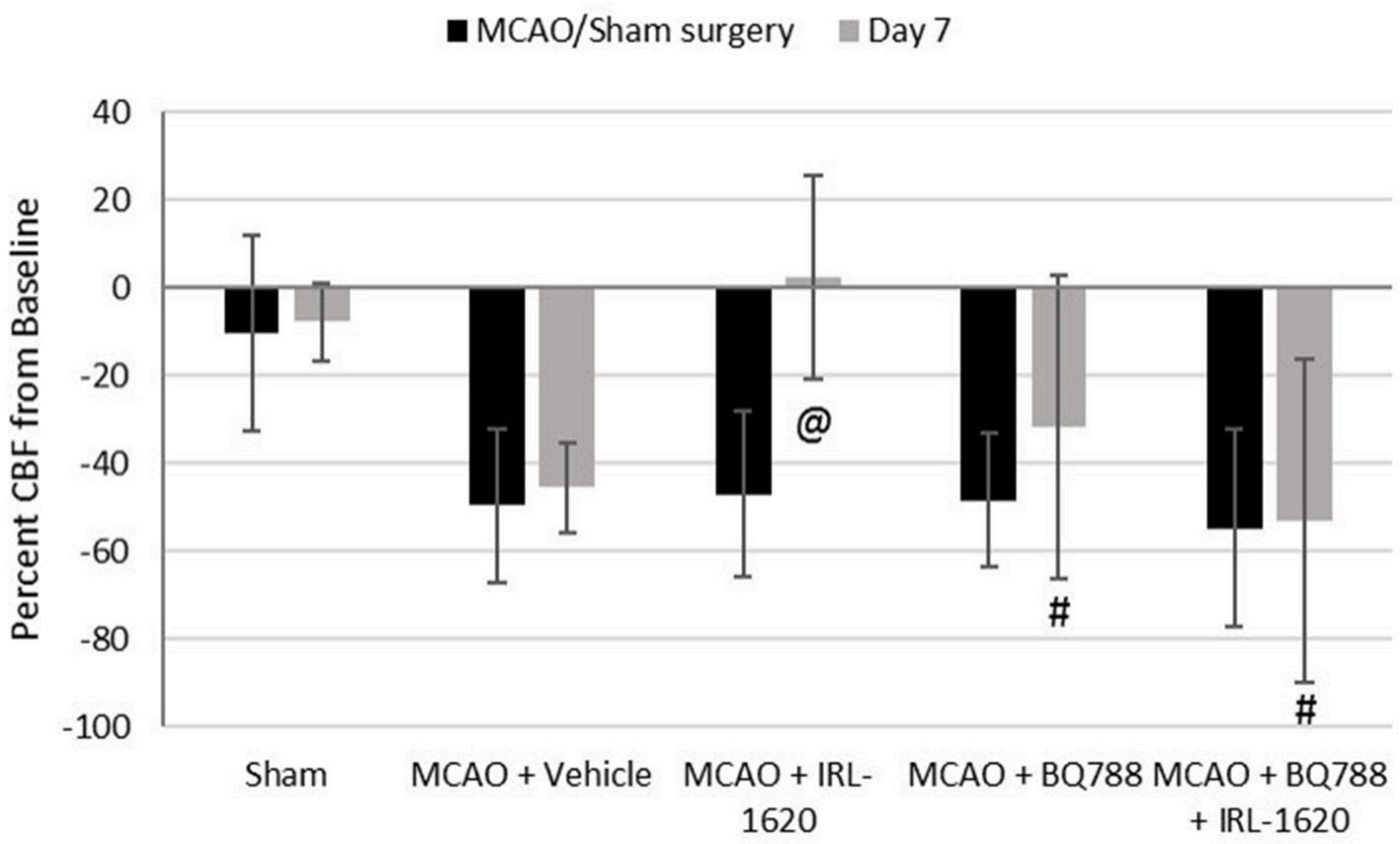
Percent change in cerebral blood flow (CBF). Treatment with IRL-1620 lessens the reduction in CBF after middle cerebral artery occlusion (MCAO). This effect was blocked by treatment with ETB antagonist BQ788. Groups I-III: *n* = 6, Group IV: *n* = 5, Group V: *n* = 4. Values are presented as mean ± SD. *P* < 0.05: @, compared to vehicle group; #, compared to IRL-1620 group.

**Figure 2:**
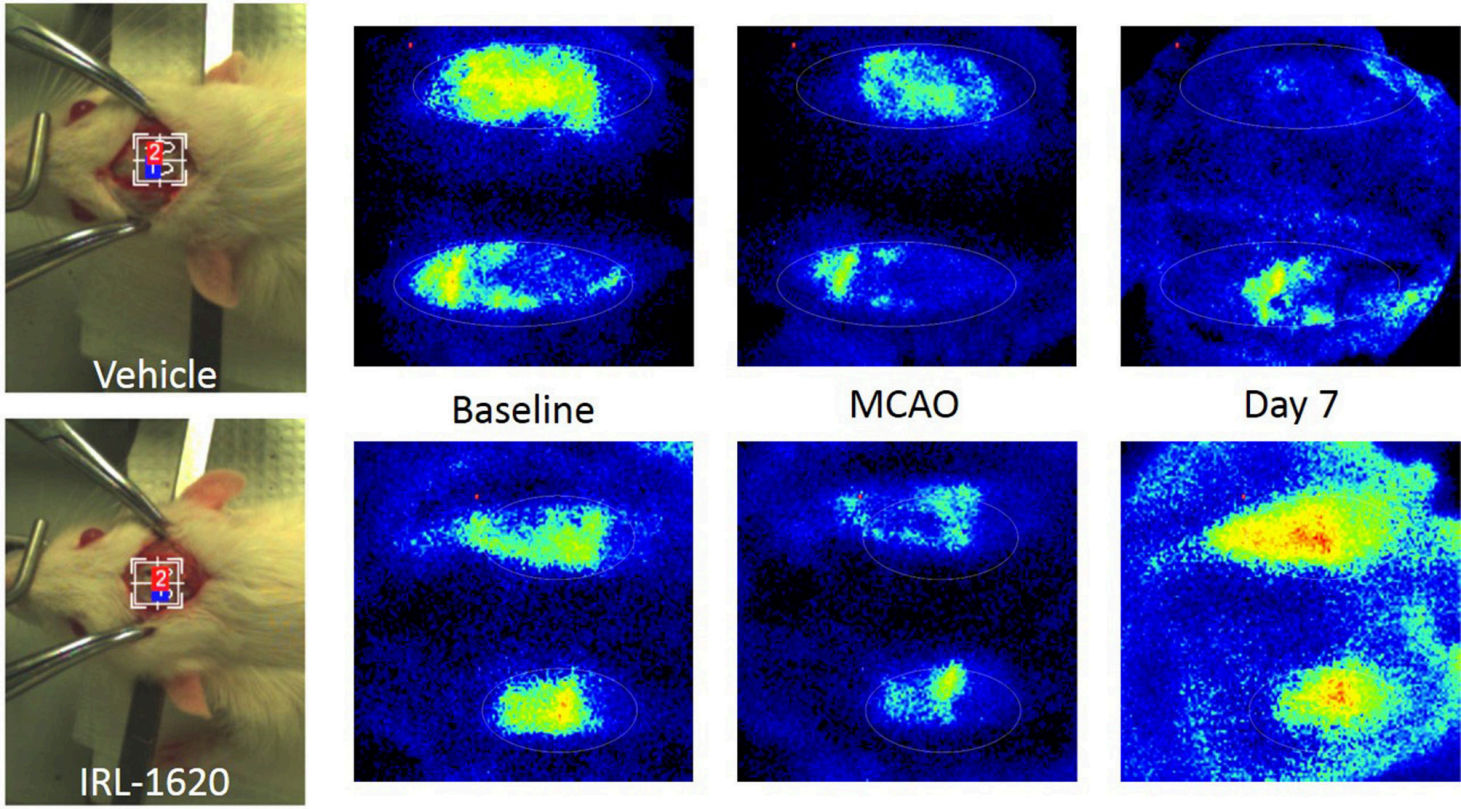
Pericam PSI Laser Speckle Contrast Analysis (LASCA) imaging of cerebral blood flow. **Top**: Group II (vehicle), left to right: brightfield image, baseline, 1 h post MCAO, 7 days post MCAO. **Bottom**: Group III (IRL-1620), left to right: brightfield image, baseline, 1 h post MCAO, 7 days post MCAO.

**Figure 3:**
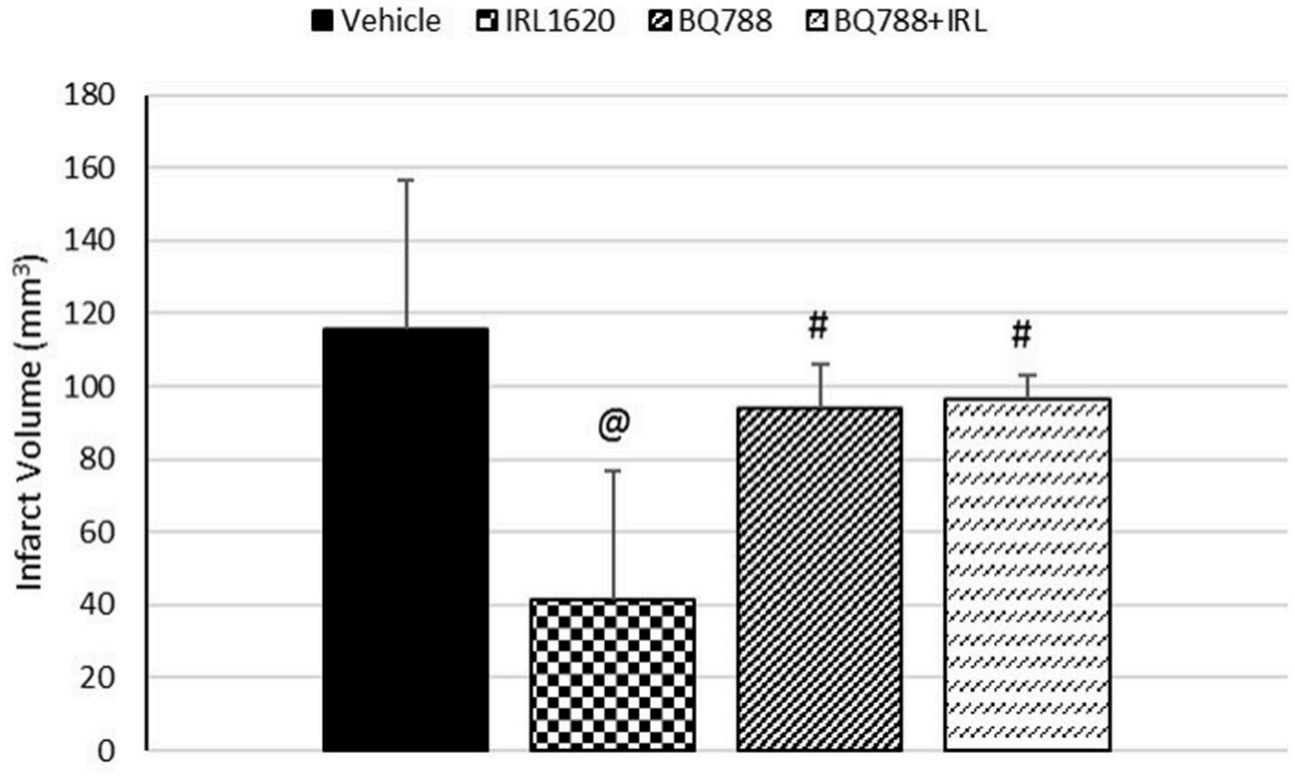
Effect of IRL-1620 and BQ788 on infarct volume (mm^3^). Groups I-III: *n* = 6, Group IV: *n* = 5, Group V: *n* = 4. Values are presented as mean ± SD. *P* < 0.05: @, compared to vehicle group; #, compared to IRL-1620 group.

**Figure 4:**
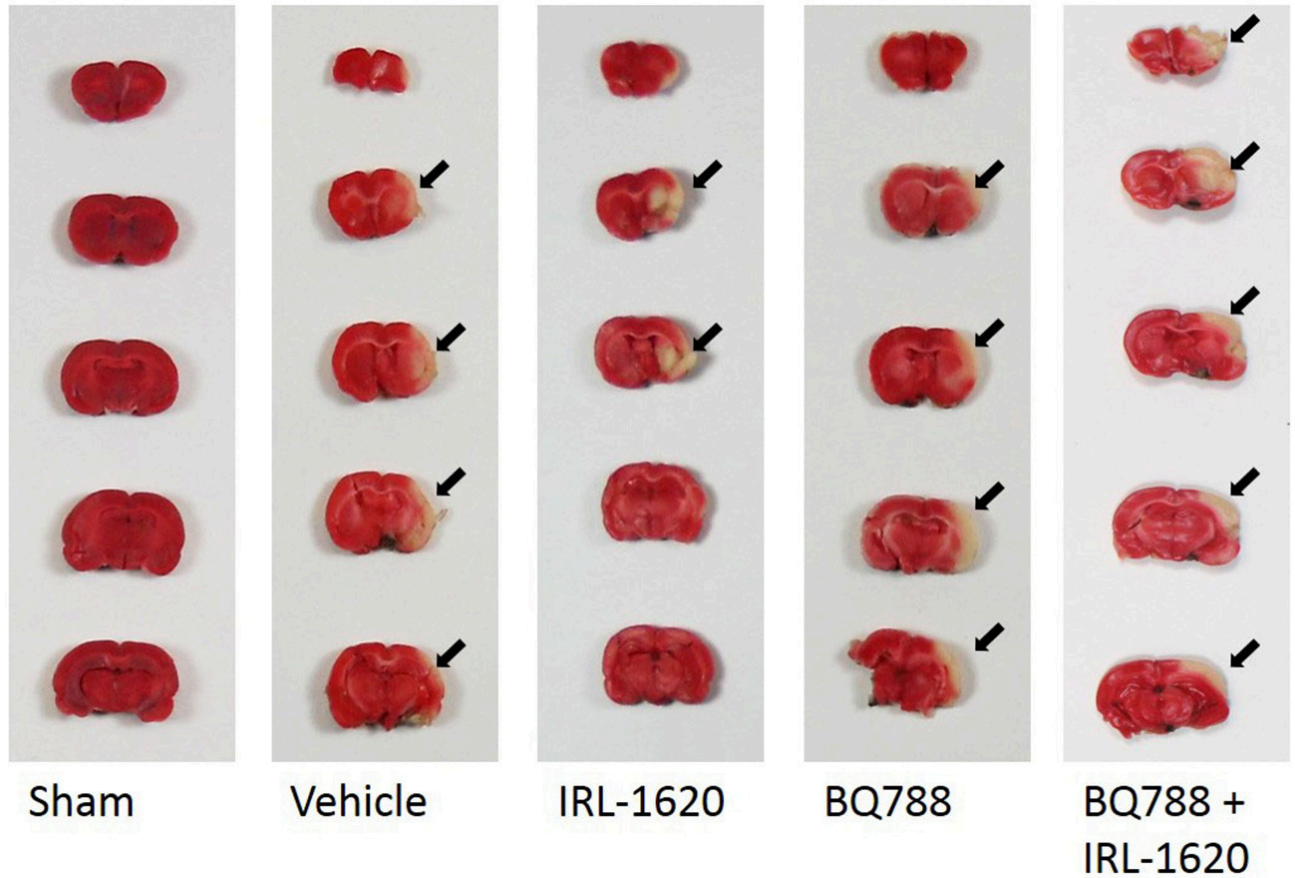
Representative images of infarct. Two-mm coronal sections of brains were stained with 2% 2,3,5-triphenyltetrazolium chloride (TTC) to visualize the infarct area. Arrows denote area of infarct. Red, normal tissue; white, infarcted tissue.

CBF is expressed as a percent of the baseline value (Figure 1). A 40–50% reduction from baseline was seen in all animals post MCAO. The IRL-1620 group experienced a significantly lower reduction in CBF (+2.3 ± 23.3%) in comparison to the vehicle- (−45.4 ± 10.2%, *p* = 0.0007), BQ877- (−31.7 ± 34.4%, *p* = 0.0304) or IRL1620-and-BQ877-treated groups (−53.1 ± 36.7%, *p* < 0.0001). Pericam images of CBF for the vehicle- and IRL-1620-treated groups are presented in Figure 2. IRL-1620-treated rats had significantly lower measurements of infarct volume in comparison to the other three treatment groups (41.4 ± 35.4 vs. 115.4 ± 40.9 mm^3^, vehicle; 93.9 ± 28.8 mm^3^, BQ788; and 96.6 ± 10.1 mm^3^, IRL-1620 + BQ788). Infarct volumes by group are presented in Figures 3, 4. While there was no mortality in Groups I-III (sham, vehicle, and IRL-1620), one animal in Group IV and two animals in Group V died prior to Day 7 assessments.

## Discussion

Our study showed that young rats that underwent MCAO and then were treated with IRL-1620 had a better recovery than vehicle-treated rats as demonstrated by a lower neurologic deficit score and better motor function at 7 days. Experimental cerebral ischemia resulted in a marked deficit in neurological function and motor performance within 24 h after MCAO, with animals demonstrating weakened or paralyzed limbs. Treatment with IRL-1620 resulted in a significant reduction in neurological deficit and motor impairment as measured by the grip test, foot-fault test, and rotarod when compared to treatment with only vehicle.

Muscle strength and coordination were significantly improved with treatment, as evidenced by an increased ability to hold on to the string in the grip test and to remain on the rotating spindle. We found no statistically significant difference in maximal recovery point for any neuro-motor tests or CBF between the IRL-1620-treated and Sham groups. Improved neuro-motor performance testing, greater CBF, and reduced infarct volume in the IRL-1620-treated group compared to the vehicle- and BQ877-treated groups supports the hypothesis that IRL-1620 plays a neuroprotective and regenerative role in ischemic role. Antagonizing the ET_B_ receptor with BQ788 blocked the positive effects of IRL-1620 treatment, resulting in deficits similar or greater than those seen in vehicle-treated rats.

The CBF in the infarcted hemisphere improved at seven days in the animals treated with IRL-1620 as compared to those treated with only vehicle (+2.3 ± 23.3% vs. −45.4 ± 10.2%), and the infarct volume was smaller (41.4 ± 35.4 mm^3^ vs. 115.4 ± 40.9 mm^3^). This demonstrates that treatment with IRL-1620 promoted recovery from stroke in our pediatric animal model. These findings are consistent with those in adult rats and published by our group previously (12, 13).

The purpose of this study was to determine the effect of selective ET_B_ receptor activation with IRL-1620 on physiological and functional recovery following an MCAO in young rats. Ontological studies have demonstrated the integral function of ET_B_ receptors within the developing brain, promoting neuronal proliferation and migration as well as regulating angiogenic growth factors (23–25). Later in life, similarly, stimulation of ET_B_ receptors following central nervous system (CNS) damage has been shown in several pre-clinical studies to enhance neurogenesis and angiogenesis, thereby promoting CNS repair mechanisms (14, 26). To verify the involvement of ET_B_ receptors in cerebral ischemia and that the effects of IRL-1620 were specific to activation of these receptors, the selective antagonist of ET_B_ receptor BQ788 was used alone and in combination with IRL-1620. However, with the paucity of new treatments for pediatric stroke, we turned to previous animal studies suggesting that IRL-1620 may improve remodeling of cerebral architecture after a stroke as a starting point for this study.

Although the effects of IRL-1620 are not clearly understood, ET_B_ enhances angiogenesis and neurogenesis and stimulates CBF, which will ultimately improve outcome after a stroke. Research done by our group has shown that IRL-1620 upregulates ET_B_ receptors and increases vascular endothelial growth factor (VEGF) in the brains of both normal neonates and adult rats subjected to cerebral ischemia (25). Early treatment with a selective ET_B_ receptor agonist may enhance VEGF and neuroprotection, thereby enabling the brains of infants suffering from hypoxic damage to repair this neuronal injury. From the microvascular perspective, activation of ET_B_ receptors with IRL-1620 is known to cause cerebral vasodilatation and increased blood flow through the release of NO (27–29).

As of today, no well-established standard treatment for stroke in the pediatric age group exists. The only treatment recommended is the administration of tissue plasminogen activator within 4.5 h after the onset of the thrombotic stroke (30). The present study demonstrated that ET_B_ receptor activation may be an innovative neuroprotective therapy for focal ischemic stroke in children, consistent with findings in adult rats, as evidenced by clinical performance, infarct volume, and oxidative stress parameters (13).

This study had several limitations. One limitation was that only thromboembolic stroke was included. The effects of IRL-1620 in the hemorrhagic and diffuse ischemic stroke should also be investigated. Similarly, like other drug interventions for stroke, the administration time window of IRL-1620 is limited to the first 6 h of the onset of the stroke event, here MCAO. Further studies using IRL-1620 after 6 h of the stroke event need to be performed to explore the potential of this drug. Another limitation was that imaging studies used in clinical practice, like computed tomography or magnetic resonance imaging, were not used to assess the stroke magnitude in the rats. However, the Pericam PSI System, a real-time microcirculation imaging device, provided a good visual assessment of the cerebral tissue blood perfusion. It also illustrated clearly the differences in regional blood flow between the infarcted hemisphere and the intact hemisphere, before and after treatment with IRL-1620 over time and at 1 h and 7 days after MCAO. Finally, there may be limitations with regards to how well this model relates to certain pediatric populations. Rats of 3 months of age were chosen to represent a population which can range from the equivalent 8–16 human years (16). Translation of these results into younger, pre-pubescent populations will require further research using a younger model.

## Conclusion

In conclusion, the ET_B_ agonist, IRL-1620, significantly reduced neurological and motor deficit as well as infarct volume while increasing CBF in a pediatric rat model of cerebral ischemia. These results suggest that selective ET_B_ receptor stimulation may provide a novel neuroprotective therapeutic strategy in the treatment of pediatric ischemic stroke.

## Author's note

AG is the director of the Laboratory at Midwestern University College of Pharmacy.

## Author contributions

All authors listed have made a substantial, direct and intellectual contribution to the work, and approved it for publication.

### Conflict of interest statement

The authors declare that the research was conducted in the absence of any commercial or financial relationships that could be construed as a potential conflict of interest.

## References

[1] Tsze DS Valente JH. Pediatric stroke: a review. Emerg Med Int. (2011) 2011:734506. 10.1155/2011/734506 22254140 PMC3255104

[2] Titomanlio L Zanin A Sachs P Khaled J Elmaleh M Blanc R. Pediatric ischemic stroke: acute management and areas of research. J Pediatr. (2013) 162:227.e1–35.e1. 10.1016/j.jpeds.2012.09.018 23153863

[3] Numis AL Fox CK. Arterial ischemic stroke in children: risk factors and etiologies. Curr Neurol Neurosci Rep. (2014) 14:422. 10.1007/s11910-013-0422-8 24384876 PMC3954544

[4] Pezzati M Filippi L Chiti G Dani C Rossi S Bertini G. Central venous catheters and cardiac tamponade in preterm infants. Intensive Care Med. (2004) 30:2253–6. 10.1007/s00134-004-2472-5 15517163

[5] Sol JJ van de Loo M Boerma M4 Bergman KA Donker AE van der Hoeven MAHBM. NEOnatal central-venous line observational study on thrombosis (NEOCLOT): evaluation of a national guideline on management of neonatal catheter-related thrombosis. BMC Pediatr. (2018) 18:84. 10.1186/s12887-018-1000-7 29475450 PMC5824541

[6] Takahashi K Ghatei MA Jones PM Murphy JK Lam HC O'Halloran DJ. Endothelin in human brain and pituitary gland: comparison with rat. J Cardiovasc Pharmacol. (1991) 17(Suppl. 7):S101–3. 10.1097/00005344-199100177-00026 1725298

[7] Rogers SD Demaster E Catton M Ghilardi JR Levin LA Maggio JE. Expression of endothelin-B receptors by glia *in vivo* is increased after CNS injury in rats, rabbits, and humans. Exp Neurol. (1997) 145:180–95. 10.1006/exnr.1997.6468 9184120

[8] Schinelli S. Pharmacology and physiopathology of the brain endothelin system: an overview. Curr Med Chem. (2006) 13:627–38. 10.2174/092986706776055652 16529555

[9] Koyama Y Michinaga S. Regulations of astrocytic functions by endothelins: roles in the pathophysiological responses of damaged brains. J Pharmacol Sci. (2012) 118:401–7. 10.1254/jphs.11R13CP 22447302

[10] Morton AJ Davenport AP. Cerebellar neurons and glia respond differentially to endothelins and sarafotoxin S6b. Brain Res. (1992) 581:299–306. 10.1016/0006-8993(92)90721-K 1393534

[11] Lucas GA White LR Juul R Cappelen J Aasly J Edvinsson L. Relaxation of human temporal artery by endothelin ETB receptors. Peptides (1996) 17:1139–44. 10.1016/S0196-9781(96)00177-5 8959748

[12] Leonard MG Briyal S Gulati A. Endothelin B receptor agonist, IRL-1620, reduces neurological damage following permanent middle cerebral artery occlusion in rats. Brain Res. (2011) 1420:48–58. 10.1016/j.brainres.2011.08.075 21959172

[13] Leonard MG Briyal S Gulati A. Endothelin B receptor agonist, IRL-1620, provides long-term neuroprotection in cerebral ischemia in rats. Brain Res. (2012) 1464:14–23. 10.1016/j.brainres.2012.05.005 22580085

[14] Leonard MG Gulati A. Endothelin B receptor agonist, IRL-1620, enhances angiogenesis and neurogenesis following cerebral ischemia in rats. Brain Res. (2013) 1528:28–41. 10.1016/j.brainres.2013.07.002 23850649

[15] Gulati A Hornick MG Briyal S Lavhale MS. A novel neuroregenerative approach using ET(B) receptor agonist, IRL-1620, to treat CNS disorders. Physiol Res. (2018) 67:S95–1123. Available online at: www.biomed.cas.cz/physiolres 29947531 10.33549/physiolres.933859

[16] Sengupta P. The laboratory rat: relating its age with human's. Int J Prev Med. (2013) 4:624–30. Available online at: www.ijpvmjournal.net 23930179 PMC3733029

[17] Koizumi J Yoshida Y Nakazawa T Ooneda G Experimental studies of ischemic brain edema. I A new experimental model of cerebral embolism in rats in which recirculation can be introduced in the ischemic area. Jpn J Stroke (1986) 8:1–8. 10.3995/jstroke.8.1

[18] Tatlisumak T Carano RA Takano K Opgenorth TJ Sotak CH Fisher M. A novel endothelin antagonist, A-127722, attenuates ischemic lesion size in rats with temporary middle cerebral artery occlusion: a diffusion and perfusion MRI study. Stroke (1998) 29:850–7; discussion: 857–8. 10.1161/01.STR.29.4.850 9550522

[19] Moran PM Higgins LS Cordell B Moser PC. Age-related learning deficits in transgenic mice expressing the 751-amino acid isoform of human beta-amyloid precursor protein. Proc Natl Acad Sci USA. (1995) 92:5341–5. 10.1073/pnas.92.12.5341 7777509 PMC41690

[20] Markgraf CG Green EJ Hurwitz BE Morikawa E Dietrich WD McCabe PM. Sensorimotor and cognitive consequences of middle cerebral artery occlusion in rats. Brain Res. (1992) 575:238–246. 10.1016/0006-8993(92)90085-N 1571783

[21] Rogers DC Campbell CA Stretton JL Mackay KB. Correlation between motor impairment and infarct volume after permanent and transient middle cerebral artery occlusion in the rat. Stroke (1997) 28:2060–65; discussion: 2066. 10.1161/01.STR.28.10.2060 9341719

[22] Barone FC White RF Elliott JD Feuerstein GZ Ohlstein EH. The endothelin receptor antagonist SB 217242 reduces cerebral focal ischemic brain injury. J Cardiovasc Pharmacol. (1995) 26(Suppl. 3):S404–7. 10.1097/00005344-199506263-00119 8587428

[23] Ehrenreich H Nau TR Dembowski C Hasselblatt M Barth M Hahn A. Endothelin b receptor deficiency is associated with an increased rate of neuronal apoptosis in the dentate gyrus. Neuroscience (2000) 95:993–1001. 10.1016/S0306-4522(99)00507-2 10682706

[24] Vidovic M Chen MM Lu QY Kalloniatis KF Martin BM Tan AH. Deficiency in endothelin receptor B reduces proliferation of neuronal progenitors and increases apoptosis in postnatal rat cerebellum. Cell Mol Neurobiol. (2008) 28:1129–38. 10.1007/s10571-008-9292-z 18683040 PMC11515047

[25] Leonard MG Prazad P Puppala B Gulati A. Selective endothelin-B receptor stimulation increases vascular endothelial growth factor in the rat brain during postnatal development. Drug Res. (2015) 65:607–13. 10.1055/s-0034-1398688 25806822

[26] Briyal S Nguyen C Leonard M Gulati A. Stimulation of endothelin B receptors by IRL-1620 decreases the progression of Alzheimer's disease. Neuroscience (2015) 301:1–11. 10.1016/j.neuroscience.2015.05.044 26022359

[27] Kobari M Fukuuchi Y Tomita M Tanahashi N Konno S Takeda H. Dilatation of cerebral microvessels mediated by endothelin ETB receptor and nitric oxide in cats. Neurosci Lett. (1994) 176:157–60. 10.1016/0304-3940(94)90071-X 7830937

[28] Kitazono T Heistad DD Faraci FM. Enhanced responses of the basilar artery to activation of endothelin-B receptors in stroke-prone spontaneously hypertensive rats. Hypertension (1995) 25:490–4. 10.1161/01.HYP.25.4.490 7721388

[29] Leonard MG Gulati A. Repeated administration of ET(B) receptor agonist, IRL-1620, produces tachyphylaxis only to its hypotensive effect. Pharmacol Res. (2009) 60:402–10. 10.1016/j.phrs.2009.07.015 19666119

[30] Rivkin MJ, Bernard TJ, Dowling MM, Amlie-Lefond C. Guidelines for urgent management of stroke in children. Pediatr Neurol. (2016) 56:8–17. 10.1016/j.pediatrneurol.2016.01.016 26969237

